# Supplementary material for: Optimized tDR Sequencing Reveals Diversity and Heterogeneity in tRNA-Derived Fragment Landscapes in Mouse Tissues
Source: Int J Mol Sci. 2025 Sep 9;26(18):8772. doi: 10.3390/ijms26188772 (PMC12469748; doi:10.3390/ijms26188772)
Supplement: Supplementary file 1 [file ijms-26-08772-s001.zip › ijms-3831360 Supplementary.pdf]

Supplementary figures

Figure S1 (supplement to Figure 3): A-D: Volcano plot of differentially expressed ntDRs in the tissue vs all differential expression analysis. **E-I:** Volcano plot of differentially expressed mtDRs in the tissue vs all differential expression analysis.

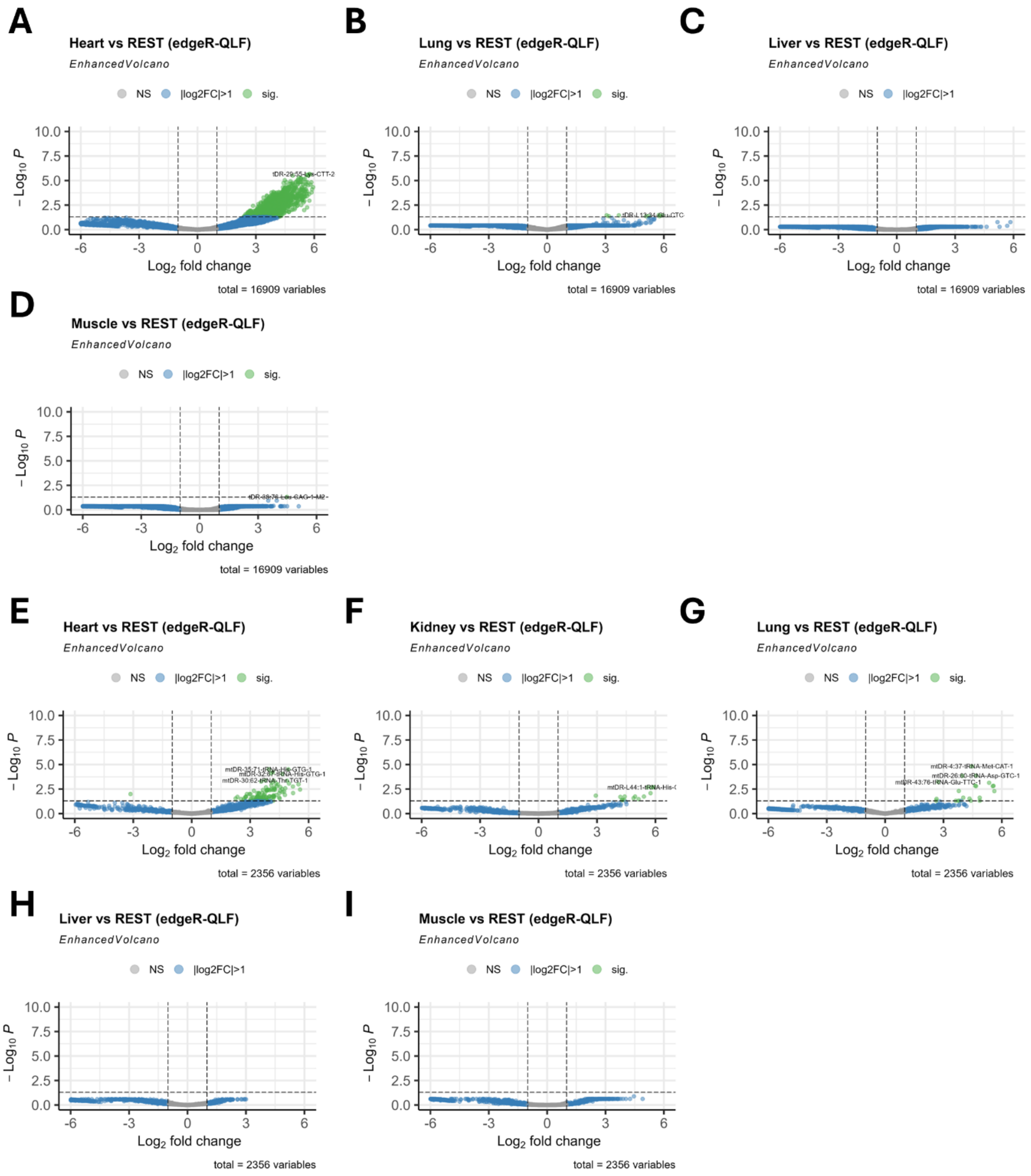
A
Heart vs REST (edgeR-QLF)
EnhancedVolcano
NS |log2FC|>1 sig.
Log2 fold change
total = 16909 variables
B
Lung vs REST (edgeR-QLF)
EnhancedVolcano
NS |log2FC|>1 sig.
Log2 fold change
total = 16909 variables
C
Liver vs REST (edgeR-QLF)
EnhancedVolcano
NS |log2FC|>1
Log2 fold change
total = 16909 variables
D
Muscle vs REST (edgeR-QLF)
EnhancedVolcano
NS |log2FC|>1 sig.
Log2 fold change
total = 16909 variables
E
Heart vs REST (edgeR-QLF)
EnhancedVolcano
NS |log2FC|>1 sig.
Log2 fold change
total = 2356 variables
F
Kidney vs REST (edgeR-QLF)
EnhancedVolcano
NS |log2FC|>1 sig.
Log2 fold change
total = 2356 variables
G
Lung vs REST (edgeR-QLF)
EnhancedVolcano
NS |log2FC|>1 sig.
Log2 fold change
total = 2356 variables
H
Liver vs REST (edgeR-QLF)
EnhancedVolcano
NS |log2FC|>1
Log2 fold change
total = 2356 variables
I
Muscle vs REST (edgeR-QLF)
EnhancedVolcano
NS |log2FC|>1 sig.
Log2 fold change
total = 2356 variables

Figure S2: Pairwise analysis of tissue-vs-tissue followed by quasi-likelihood (QL) post-hoc analysis to identify tDRs patterns. **A:** summary of statistically significant ntDRs ($|log2FC| \geq 1$, $FDR < 0.05$) in all pairwise comparisons. The spleen was the most diverse tissue with the highest number of significant ntDRs in all comparisons. **B:** Heatmap of top ntDRs detected by omnibus QL F-test across all pairwise comparisons. Note that the spleen and lung samples cluster closely. **C:** summary of statistically significant mtDRs ($|log2FC| \geq 1$, $FDR < 0.05$) in all pairwise comparisons. The spleen was the most diverse tissue with the highest number of significant mtDRs in all comparisons. **D:** Bar plot showing the number of tissue specific ntDRs detected by the omnibus QL F-test. The spleen had the highest number of specific ntDRs.

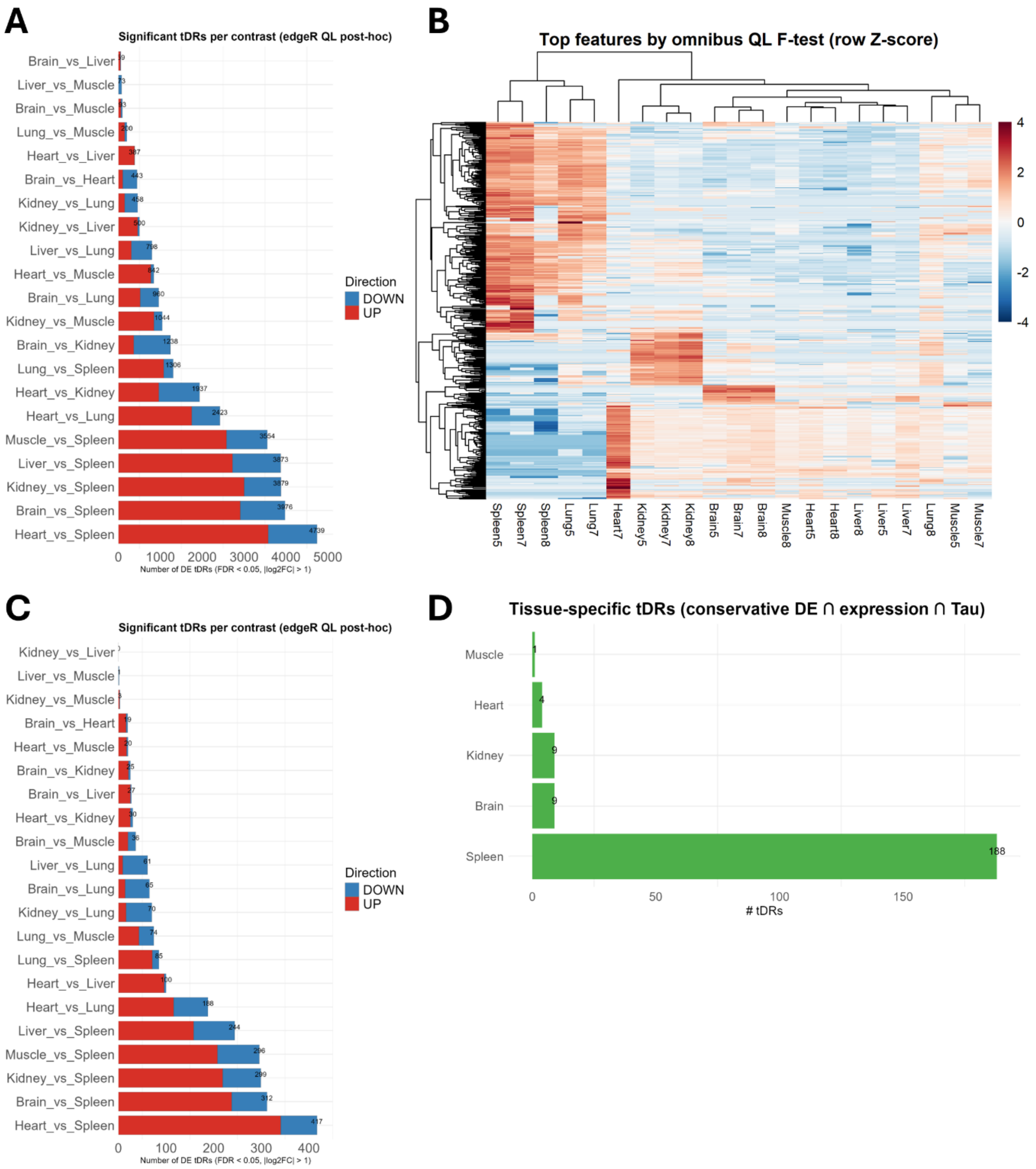
A
Significant tDRs per contrast (edgeR QL post-hoc)
Brain_vs_Liver
Liver_vs_Muscle
Brain_vs_Muscle
Lung_vs_Muscle
Heart_vs_Liver
Brain_vs_Heart
Kidney_vs_Lung
Kidney_vs_Liver
Liver_vs_Lung
Heart_vs_Muscle
Brain_vs_Lung
Kidney_vs_Muscle
Brain_vs_Kidney
Lung_vs_Spleen
Heart_vs_Kidney
Heart_vs_Lung
Muscle_vs_Spleen
Liver_vs_Spleen
Kidney_vs_Spleen
Brain_vs_Spleen
Heart_vs_Spleen
0 1000 2000 3000 4000 5000
Number of DE tDRs (FDR < 0.05, |log2FC| > 1)
Direction
DOWN
UP
B
Top features by omnibus QL F-test (row Z-score)
4
2
0
-2
-4
Spleen5 Spleen7 Spleen8 Lung5 Lung7 Heart7 Kidney5 Kidney7 Kidney8 Brain5 Brain7 Brain8 Muscle8 Heart5 Heart8 Liver8 Liver5 Liver7 Lung8 Muscle5 Muscle7
C
Significant tDRs per contrast (edgeR QL post-hoc)
Kidney_vs_Liver
Liver_vs_Muscle
Kidney_vs_Muscle
Brain_vs_Heart
Heart_vs_Muscle
Brain_vs_Kidney
Brain_vs_Liver
Heart_vs_Kidney
Brain_vs_Muscle
Liver_vs_Lung
Brain_vs_Lung
Kidney_vs_Lung
Lung_vs_Muscle
Lung_vs_Spleen
Heart_vs_Liver
Heart_vs_Lung
Liver_vs_Spleen
Muscle_vs_Spleen
Kidney_vs_Spleen
Brain_vs_Spleen
Heart_vs_Spleen
0 100 200 300 400
Number of DE tDRs (FDR < 0.05, |log2FC| > 1)
Direction
DOWN
UP
D
Tissue-specific tDRs (conservative DE ∩ expression ∩ Tau)
Muscle
Heart
Kidney
Brain
Spleen
1
4
9
9
188
0 50 100 150
tDRs

Figure S3 (supplement to figure 4). A-C: Distribution of significant ntDR subclasses in different tissues versus all other tissues. **D-F:** Distribution of significant mt-tDR subclasses in different tissues versus all other tissues. These bar plots represent simple counts of the number of significantly enriched tDRs in the tissue vs all analysis and their subclass membership based on our classifier.

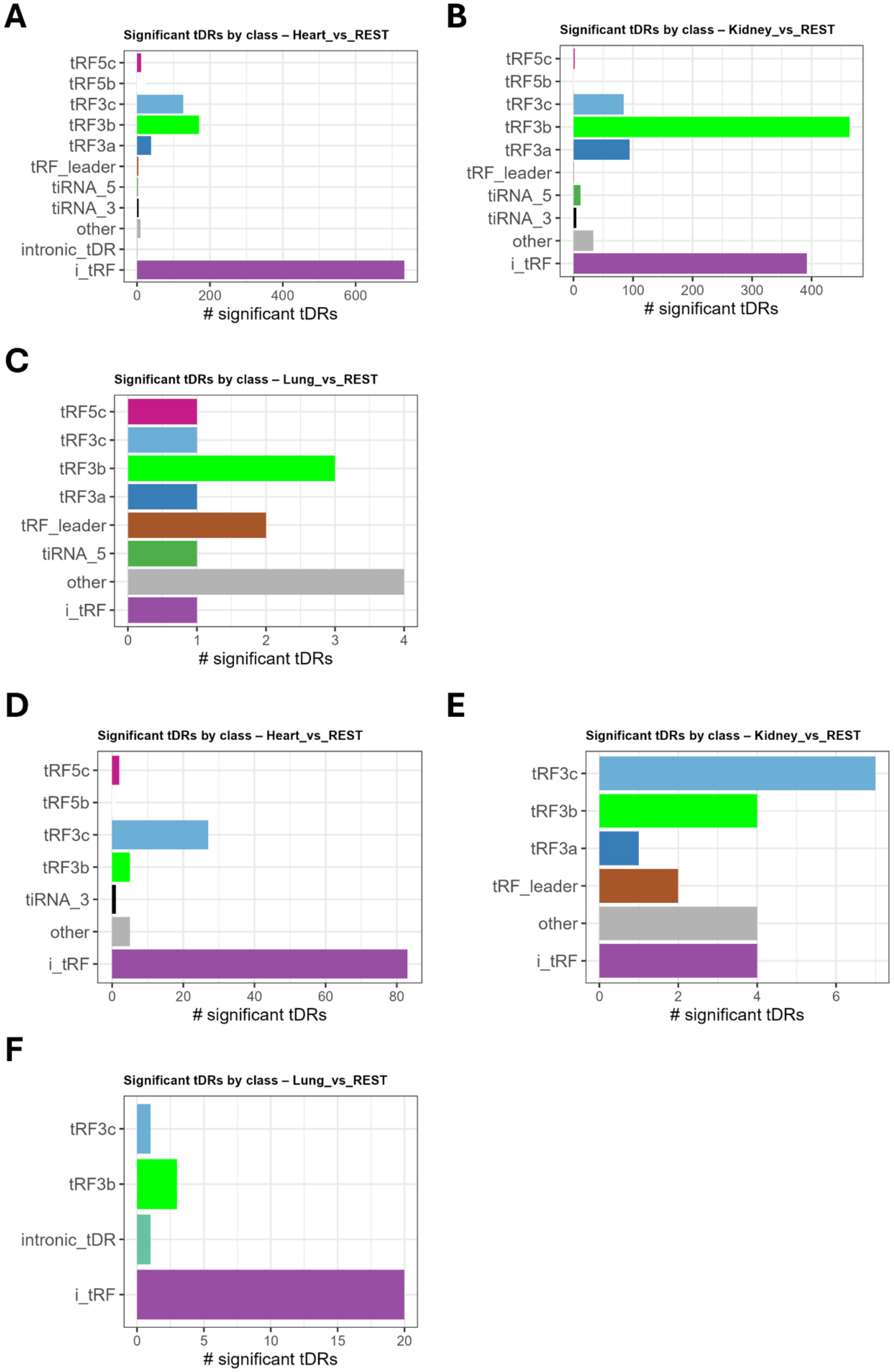
A
Significant tDRs by class – Heart_vs_REST
tRF5c
tRF5b
tRF3c
tRF3b
tRF3a
tRF_leader
tiRNA_5
tiRNA_3
other
intronic_tDR
i_tRF
0
200
400
600
significant tDRs
B
Significant tDRs by class – Kidney_vs_REST
tRF5c
tRF5b
tRF3c
tRF3b
tRF3a
tRF_leader
tiRNA_5
tiRNA_3
other
i_tRF
0
100
200
300
400
significant tDRs
C
Significant tDRs by class – Lung_vs_REST
tRF5c
tRF3c
tRF3b
tRF3a
tRF_leader
tiRNA_5
other
i_tRF
0
1
2
3
4
significant tDRs
D
Significant tDRs by class – Heart_vs_REST
tRF5c
tRF5b
tRF3c
tRF3b
tiRNA_3
other
i_tRF
0
20
40
60
80
significant tDRs
E
Significant tDRs by class – Kidney_vs_REST
tRF3c
tRF3b
tRF3a
tRF_leader
other
i_tRF
0
2
4
6
significant tDRs
F
Significant tDRs by class – Lung_vs_REST
tRF3c
tRF3b
intronic_tDR
i_tRF
0
5
10
15
20
significant tDRs

Figure S4: A: Density plot of size distribution of the upregulated mtDRs in tissue vs all comparison. **B:** Heatmap of Kolmogorov-Smirnov statistical analysis of length distribution of upregulated ntDRs in each condition. Higher values indicate statistically significant. All comparisons were statistically significant ($p < 0.05$, FDR < 0.05). C: Sprinzl heatmap showing the mapping location of significantly upregulated ntDRs across the mature tRNA. The heatmap reveals the heterogeneity in tDR subclasses as well as the size distribution differences between tissues.

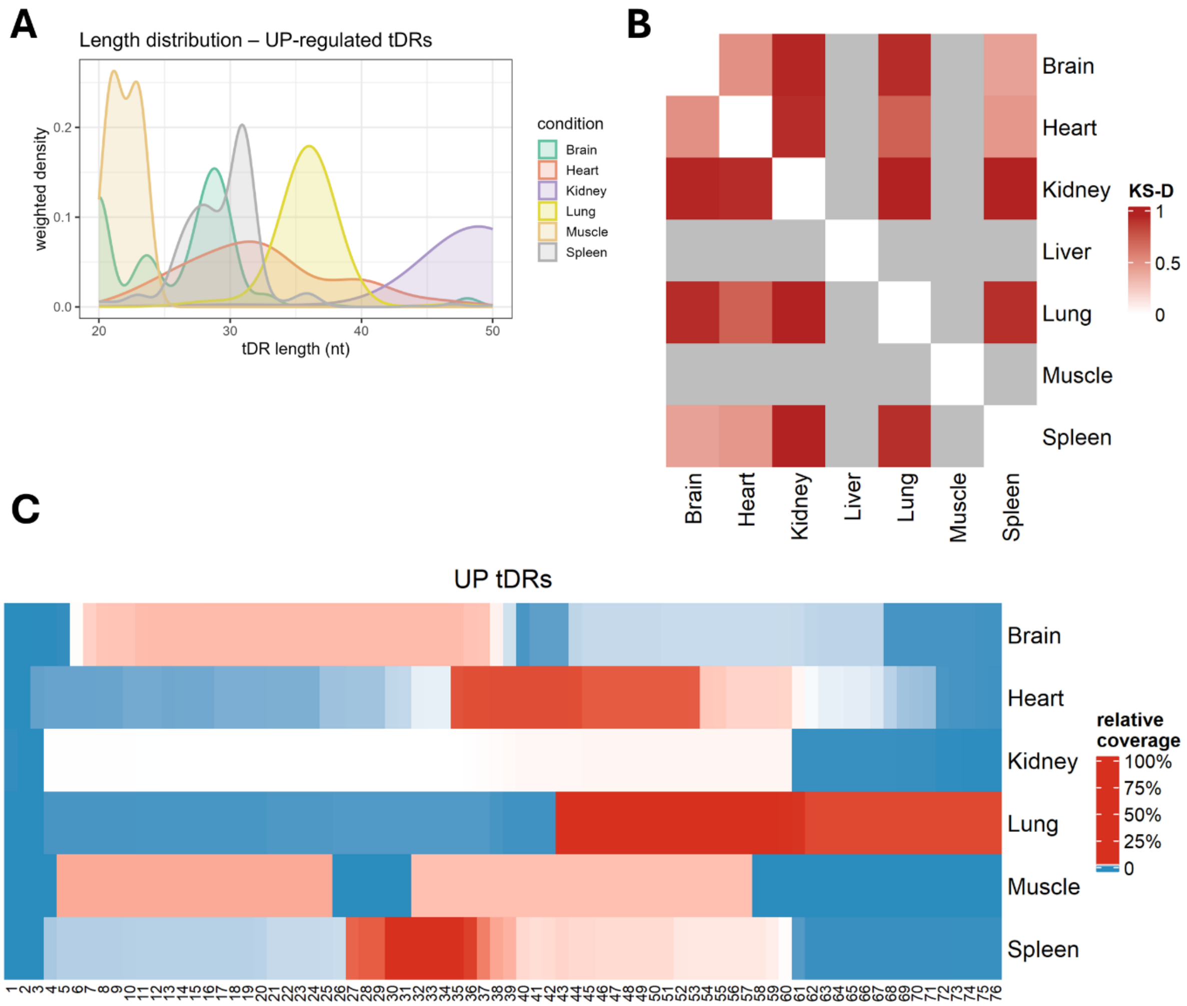
A
Length distribution – UP-regulated tDRs
weighted density
0.0
0.1
0.2
20
30
40
50
tDR length (nt)
condition
Brain
Heart
Kidney
Lung
Muscle
Spleen
B
Brain
Heart
Kidney
Liver
Lung
Muscle
Spleen
KS-D
1
0.5
0
C
UP tDRs
Brain
Heart
Kidney
Lung
Muscle
Spleen
relative coverage
100%
75%
50%
25%
0

Figure S5 (supplement to figure 5C): Dot plot of log2 odds ratio of an isodecoder tRNA to be the origin of ntDRs in different tissues. The size of the bubble reflects the number of enriched ntDRs mapped to this isodecoder.

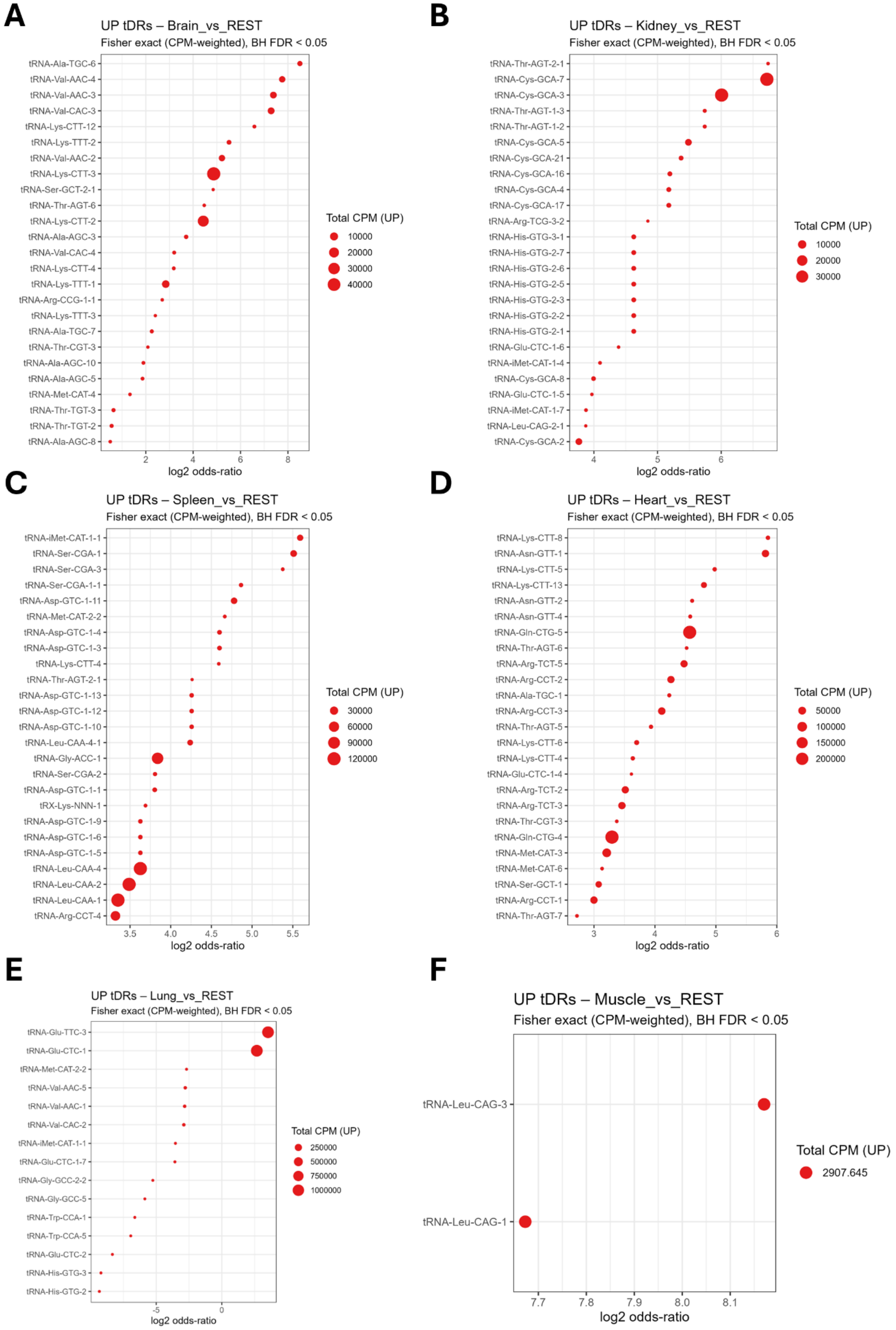
A
UP tDRs – Brain_vs_REST
Fisher exact (CPM-weighted), BH FDR < 0.05
tRNA-Ala-TGC-6
tRNA-Val-AAC-4
tRNA-Val-AAC-3
tRNA-Val-CAC-3
tRNA-Lys-CTT-12
tRNA-Lys-TTT-2
tRNA-Val-AAC-2
tRNA-Lys-CTT-3
tRNA-Ser-GCT-2-1
tRNA-Thr-AGT-6
tRNA-Lys-CTT-2
tRNA-Ala-AGC-3
tRNA-Val-CAC-4
tRNA-Lys-CTT-4
tRNA-Lys-TTT-1
tRNA-Arg-CCG-1-1
tRNA-Lys-TTT-3
tRNA-Ala-TGC-7
tRNA-Thr-CGT-3
tRNA-Ala-AGC-10
tRNA-Ala-AGC-5
tRNA-Met-CAT-4
tRNA-Thr-TGT-3
tRNA-Thr-TGT-2
tRNA-Ala-AGC-8
2
4
6
8
log2 odds-ratio
Total CPM (UP)
10000
20000
30000
40000
B
UP tDRs – Kidney_vs_REST
Fisher exact (CPM-weighted), BH FDR < 0.05
tRNA-Thr-AGT-2-1
tRNA-Cys-GCA-7
tRNA-Cys-GCA-3
tRNA-Thr-AGT-1-3
tRNA-Thr-AGT-1-2
tRNA-Cys-GCA-5
tRNA-Cys-GCA-21
tRNA-Cys-GCA-16
tRNA-Cys-GCA-4
tRNA-Cys-GCA-17
tRNA-Arg-TCG-3-2
tRNA-His-GTG-3-1
tRNA-His-GTG-2-7
tRNA-His-GTG-2-6
tRNA-His-GTG-2-5
tRNA-His-GTG-2-3
tRNA-His-GTG-2-2
tRNA-His-GTG-2-1
tRNA-Glu-CTC-1-6
tRNA-iMet-CAT-1-4
tRNA-Cys-GCA-8
tRNA-Glu-CTC-1-5
tRNA-iMet-CAT-1-7
tRNA-Leu-CAG-2-1
tRNA-Cys-GCA-2
4
5
6
log2 odds-ratio
Total CPM (UP)
10000
20000
30000
C
UP tDRs – Spleen_vs_REST
Fisher exact (CPM-weighted), BH FDR < 0.05
tRNA-iMet-CAT-1-1
tRNA-Ser-CGA-1
tRNA-Ser-CGA-3
tRNA-Ser-CGA-1-1
tRNA-Asp-GTC-1-11
tRNA-Met-CAT-2-2
tRNA-Asp-GTC-1-4
tRNA-Asp-GTC-1-3
tRNA-Lys-CTT-4
tRNA-Thr-AGT-2-1
tRNA-Asp-GTC-1-13
tRNA-Asp-GTC-1-12
tRNA-Asp-GTC-1-10
tRNA-Leu-CAA-4-1
tRNA-Gly-ACC-1
tRNA-Ser-CGA-2
tRNA-Asp-GTC-1-1
tRX-Lys-NNN-1
tRNA-Asp-GTC-1-9
tRNA-Asp-GTC-1-6
tRNA-Asp-GTC-1-5
tRNA-Leu-CAA-4
tRNA-Leu-CAA-2
tRNA-Leu-CAA-1
tRNA-Arg-CCT-4
3.5
4.0
4.5
5.0
5.5
log2 odds-ratio
Total CPM (UP)
30000
60000
90000
120000
D
UP tDRs – Heart_vs_REST
Fisher exact (CPM-weighted), BH FDR < 0.05
tRNA-Lys-CTT-8
tRNA-Asn-GTT-1
tRNA-Lys-CTT-5
tRNA-Lys-CTT-13
tRNA-Asn-GTT-2
tRNA-Asn-GTT-4
tRNA-Gln-CTG-5
tRNA-Thr-AGT-6
tRNA-Arg-TCT-5
tRNA-Arg-CCT-2
tRNA-Ala-TGC-1
tRNA-Arg-CCT-3
tRNA-Thr-AGT-5
tRNA-Lys-CTT-6
tRNA-Lys-CTT-4
tRNA-Glu-CTC-1-4
tRNA-Arg-TCT-2
tRNA-Arg-TCT-3
tRNA-Thr-CGT-3
tRNA-Gln-CTG-4
tRNA-Met-CAT-3
tRNA-Met-CAT-6
tRNA-Ser-GCT-1
tRNA-Arg-CCT-1
tRNA-Thr-AGT-7
3
4
5
6
log2 odds-ratio
Total CPM (UP)
50000
100000
150000
200000
E
UP tDRs – Lung_vs_REST
Fisher exact (CPM-weighted), BH FDR < 0.05
tRNA-Glu-TTC-3
tRNA-Glu-CTC-1
tRNA-Met-CAT-2-2
tRNA-Val-AAC-5
tRNA-Val-AAC-1
tRNA-Val-CAC-2
tRNA-iMet-CAT-1-1
tRNA-Glu-CTC-1-7
tRNA-Gly-GCC-2-2
tRNA-Gly-GCC-5
tRNA-Trp-CCA-1
tRNA-Trp-CCA-5
tRNA-Glu-CTC-2
tRNA-His-GTG-3
tRNA-His-GTG-2
-5
0
log2 odds-ratio
Total CPM (UP)
250000
500000
750000
1000000
F
UP tDRs – Muscle_vs_REST
Fisher exact (CPM-weighted), BH FDR < 0.05
tRNA-Leu-CAG-3
tRNA-Leu-CAG-1
7.7
7.8
7.9
8.0
8.1
log2 odds-ratio
Total CPM (UP)
2907.645

Figure S6: Analysis of mature tRNA expression in different tissues. Here, we conduct differential mature tRNA expression analysis comparing each tissue to all other tissues (as done for tDRs) using EdgeR. Only in the brain we observed upregulation of several isodecoders that correlated with upregulated ntDRs membership. No other tissue showed such patterns or correlations.

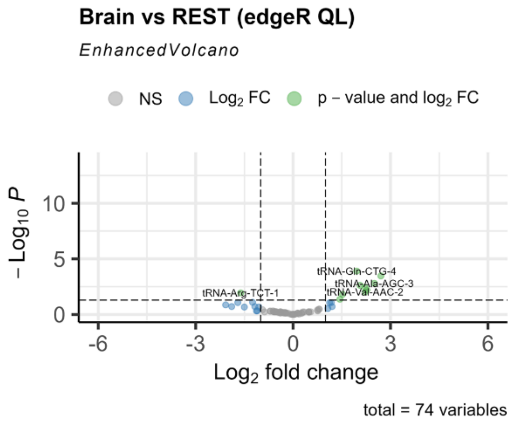
Brain vs REST (edgeR QL)
EnhancedVolcano
NS
Log_2 FC
p – value and log_2 FC
$-Log_{10}$ P
tRNA-Gln-CTG-4
tRNA-Ala-AGC-3
tRNA-Val-AAC-2
tRNA-Arg-TCT-1
Log_2 fold change
total = 74 variables

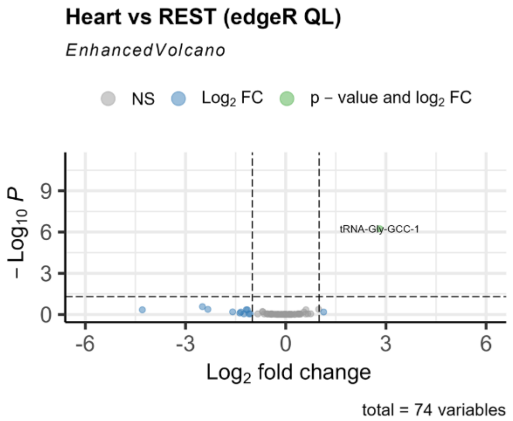
Heart vs REST (edgeR QL)
EnhancedVolcano
NS
Log_2 FC
p – value and log_2 FC
$-Log_{10}$ P
tRNA-Gly-GCC-1
Log_2 fold change
total = 74 variables

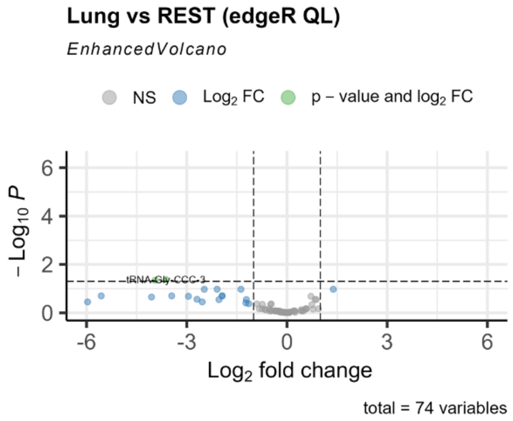
Lung vs REST (edgeR QL)
EnhancedVolcano
NS
Log_2 FC
p – value and log_2 FC
$-Log_{10}$ P
tRNA-Gly-CCC-3
Log_2 fold change
total = 74 variables

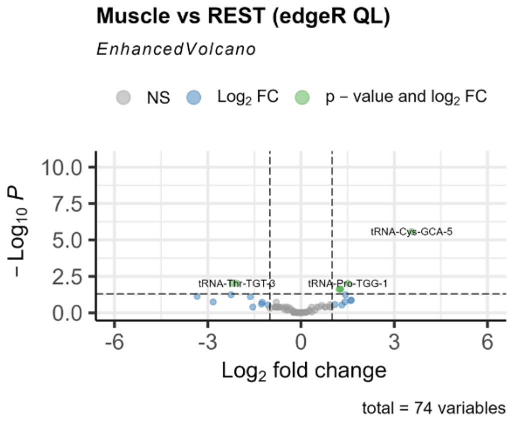
Muscle vs REST (edgeR QL)
EnhancedVolcano
NS
Log_2 FC
p – value and log_2 FC
$-Log_{10}$ P
tRNA-Cys-GCA-5
tRNA-Thr-TGT-3
tRNA-Pro-TGG-1
Log_2 fold change
total = 74 variables

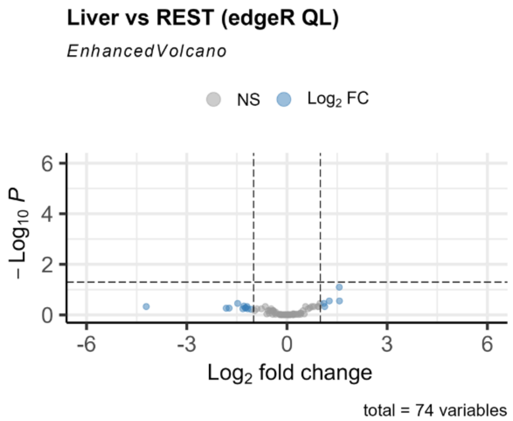
Liver vs REST (edgeR QL)
EnhancedVolcano
NS
Log_2 FC
$-Log_{10}$ P
Log_2 fold change
total = 74 variables

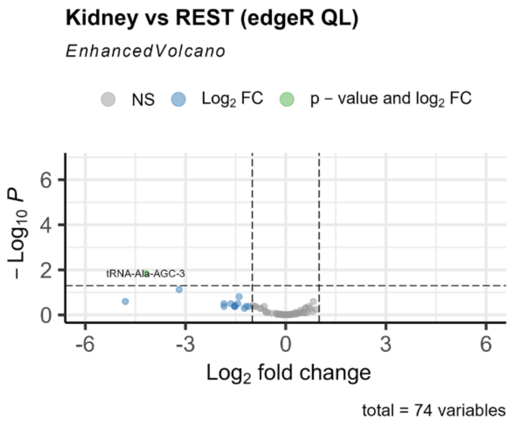
Kidney vs REST (edgeR QL)
EnhancedVolcano
NS
Log_2 FC
p – value and log_2 FC
$-Log_{10}$ P
tRNA-Ala-AGC-3
Log_2 fold change
total = 74 variables

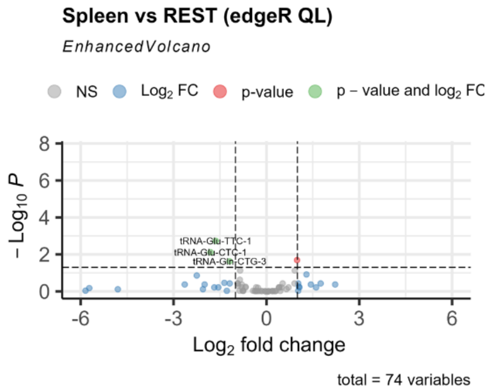
Spleen vs REST (edgeR QL)
EnhancedVolcano
NS
Log_2 FC
p-value
p – value and log_2 FC
$-Log_{10}$ P
tRNA-Glu-TTC-1
tRNA-Glu-CTC-1
tRNA-Gln-CTG-3
Log_2 fold change
total = 74 variables

Figure S7: Isotype enrichment (**A**) and Anticodon (**B**) enrichment of the top 500 ntDRs in each tissue showing variations in source tRNAs with unique signatures in the spleen and lungs. The heatmaps represent log2 Odds ratio derived from Fisher's exact test with Benjamin-Hochberg (BH) multiple test correction analysis. **C:** Heatmap of -log10 FDR of parent tRNA enrichment of the top expressed 500 ntDRs. This heatmap gives information regarding the mature tRNA isodecoder source of top enriched ntDRs.

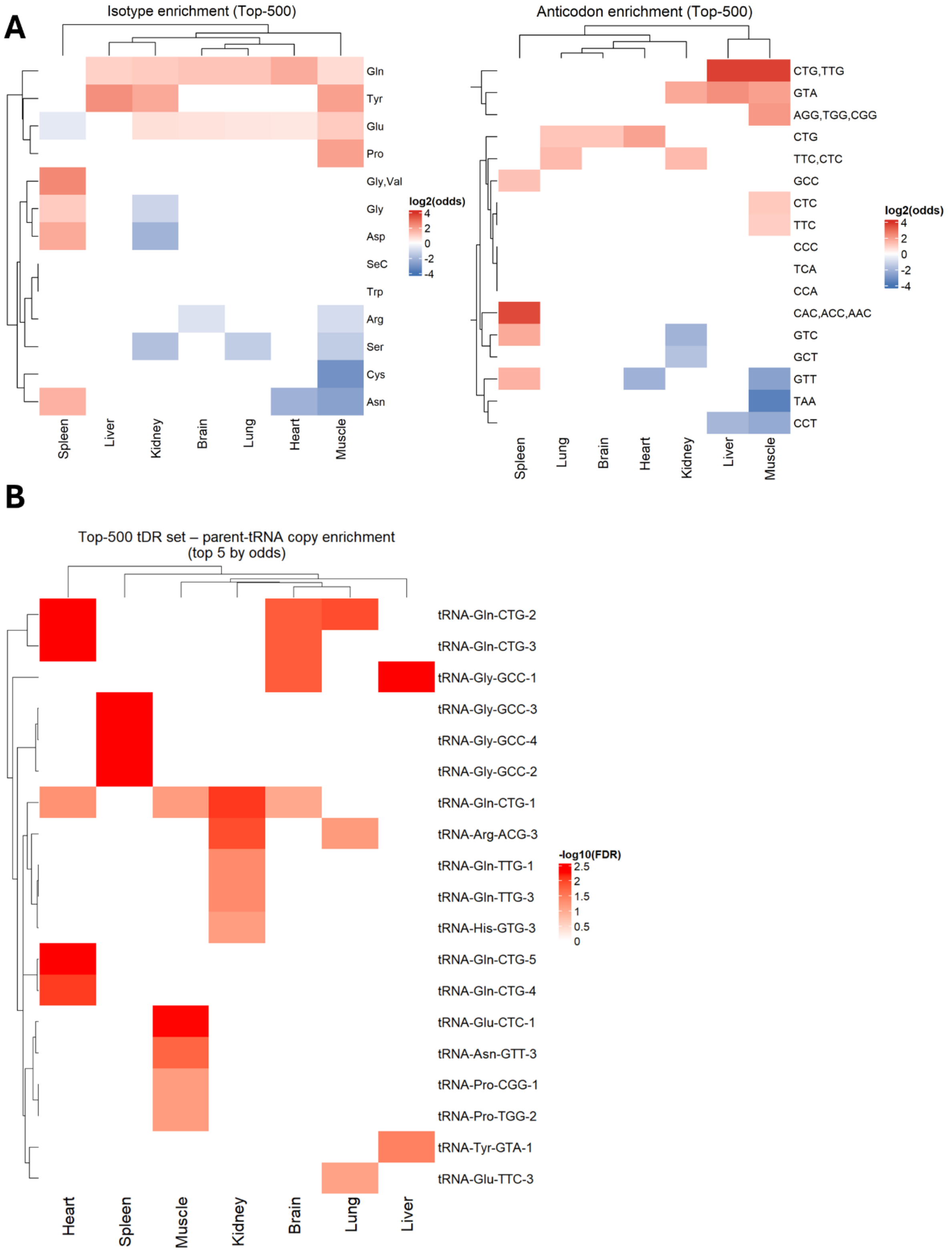
A
Isotype enrichment (Top-500)
Gln
Tyr
Glu
Pro
Gly,Val
Gly
Asp
SeC
Trp
Arg
Ser
Cys
Asn
log2(odds)
4
2
0
-2
-4
Spleen
Liver
Kidney
Brain
Lung
Heart
Muscle
Anticodon enrichment (Top-500)
CTG,TTG
GTA
AGG,TGG,CGG
CTG
TTC,CTC
GCC
CTC
TTC
CCC
TCA
CCA
CAC,ACC,AAC
GTC
GCT
GTT
TAA
CCT
log2(odds)
4
2
0
-2
-4
Spleen
Lung
Brain
Heart
Kidney
Liver
Muscle
B
Top-500 tDR set – parent-tRNA copy enrichment
(top 5 by odds)
tRNA-Gln-CTG-2
tRNA-Gln-CTG-3
tRNA-Gly-GCC-1
tRNA-Gly-GCC-3
tRNA-Gly-GCC-4
tRNA-Gly-GCC-2
tRNA-Gln-CTG-1
tRNA-Arg-ACG-3
tRNA-Gln-TTG-1
tRNA-Gln-TTG-3
tRNA-His-GTG-3
tRNA-Gln-CTG-5
tRNA-Gln-CTG-4
tRNA-Glu-CTC-1
tRNA-Asn-GTT-3
tRNA-Pro-CGG-1
tRNA-Pro-TGG-2
tRNA-Tyr-GTA-1
tRNA-Glu-TTC-3
-log10(FDR)
2.5
2
1.5
1
0.5
0
Heart
Spleen
Muscle
Kidney
Brain
Lung
Liver
